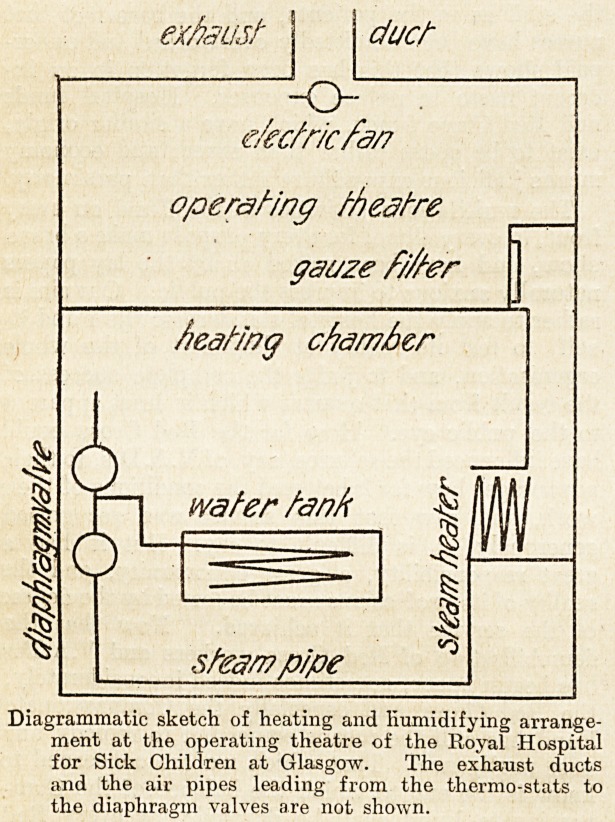# The Heating of Operating-Theatres

**Published:** 1919-05-03

**Authors:** 


### THE HEATING OF OPERATING-THEATRES.

Operating-theatres require special treatment in the matter of heating. A comparatively high temperature may be required by the operating surgeon at any moment and at any time of the year, and it is important that the theatre shall be got ready easily and quickly.

It is also of great importance that the air shall be quickly exhausted from the theatre so as to carry off all the fumes that are necessarily present; and this must be done without creating a draught or causing a chill to the patient or any one in the theatre.

In pretty well every modern hospital, the heating of the operating-theatre is arranged for quite separately from that of the wards and other parts of the building. Hot water is used in only a few cases, and even then it is assisted by steam radiators. Steam is the favourite agent employed, because by its aid the comparatively small area of the theatre can be quickly raised to any temperature, and because the temperature can be quickly lowered. Steam is generally employed, in special forms of radiators, at a pressure of 5 lb. per square inch; at that pressure there is no unpleasant smell of "burnt air," such as is often found when using higher pressures. As the quantity of steam required also is small and variable, no special apparatus is provided for collecting the water formed by its condensation, such as has been described in connection with the heating of wards; it is merely carried off to the hot well, or wherever the boiler is fed from. Where hot water is employed special arrangements are made. At the new King's College Hospital at Denmark Hill, pipes of small diameter are sunk in the plaster of the walls and ceiling, the plaster being of a special substance whose rate of expansion and contraction, with the rise and fall of temperature, is the same as that of the pipes, so that the danger of cracking the plaster is avoided. A special calorifier is employed to heat the water which circulates through the pipes, and the hot water is assisted by a battery of steam radiators working at 5 lb. per square inch.

Diagrammatic sketch of heating and humidifying arrangement at the operating theatre of the Royal Hospital for Sick Children at Glasgow. The exhaust ducts and the air pipes leading from the thermo-stats to the diaphragm valves are not shown.

The use of steam at the low pressure named enables the theatre to be heated quickly, and the air is not raised to too high a temperature. Water heats up slowly, and cools slowly; so that if it is suddenly required, the heat supplied by hot water cannot be readily lessened; but that of steam can be. Thermo-stats can quite easily control the temperature when steam at low pressure is the heating agent, but not so readily with hot water.

At the Royal Hospital for Sick Children in Glasgow, a very complete arrangement has been provided in connection with the operating-theatre for controlling both the temperature and the humidity of the air. The theatres are steam heated by a modification of the Plenum system; there is a heating chamber in the sub-basement under the theatre, in which a steam heater is fixed, in the path of the air that is being drawn up into the theatre; in addition, there is an open pan containing water, and a coil of pipe through which steam flows, lying in the pan, also in the path of the air. The heat of the steam coil causes the water to evaporate, and the air passing over the surface of the water absorbs a certain quantity of it, depending upon its own temperature, the vapour tension of the moisture already in the air, and that issuing from the water. The air, charged with moisture up to a certain humidity, is drawn into the theatre through a copper gauze filtering screen, by the aid of a fan in the theatre; and thence by means of extract air shafts, to the outer atmosphere. Both the temperature of the air and its humidity are controlled automatically from the theatre by means of diaphragm valves, worked by compressed air; the air is compressed by a small hydraulic compressor fixed in the heating chamber, and the diaphragm valves are connected by small air pipes to thermo-stats and humidi-stats, fixed in the theatre.

The temperature of the theatre is arranged for 75°F. normally, but higher temperatures up to 90°F. can be obtained if required, by altering the regulation of the thermo-stats.

The vitiated air is drawn out of the theatre by a noiseless centrifugal fan running in a chamber in the roof, to which the extract ducts mentioned above are connected; the ducts are led to different levels in the theatre, so that the air is exhausted from all parts of it.

## Figures and Tables

**Figure f1:**